# Causal Analysis Shows Evidence of Atopic Dermatitis Leading to an Increase in Vitamin D Levels

**DOI:** 10.1016/j.jid.2020.09.013

**Published:** 2021-05

**Authors:** Daniel R. Drodge, Ashley Budu-Aggrey, Lavinia Paternoster

**Affiliations:** 1 Medical Research Council Integrative Epidemiology Unit, Bristol Medical School, University of Bristol, Bristol, United Kingdom; 2 University Hospitals Bristol NHS Foundation Trust, Bristol, United Kingdom; 3 Population Health Sciences, Bristol Medical School, University of Bristol, Bristol, United Kingdom

**Keywords:** 25-OHD, 25-hydroxycholecalciferol, AD, atopic dermatitis, MR, Mendelian randomization

To the Editor

Atopic dermatitis (AD, eczema) is an inflammatory skin condition that typically presents as erythema, scaling, and urticaria (Weidinger and Novak, 2016). Prevalence in the U.S. is approximately 10% and observed to be higher in children than adults (Drucker et al., 2017). Vitamin D supplementation has been suggested for treatment, with conflicting results reported in the literature (Bath-Hextall et al., 2012; Hattangdi-Haridas et al., 2019). There is evidence of an observational association between AD and vitamin D, where sufferers, especially children, have been found to have reduced levels, with greater deficiency seen in severe AD (Palmer, 2015).

Traditional observational studies can be biased because of confounding and reverse causation. Mendelian randomization (MR) is a useful tool to investigate the presence and direction of a causal relationship. Exposure-associated SNPs are used as a proxy for the exposure of interest to investigate the causal effect on an outcome (Budu-Aggrey and Paternoster, 2019). MR has been previously used to investigate the causal effect of vitamin D (25-hydroxycholecalciferol [25-OHD]) levels on AD risk (Manousaki et al., 2017). In this study, we extend this analysis using GWAS data from the most current vitamin D meta-analysis (Manousaki et al., 2020) to identify evidence of causality and the direction of causal effect.

We investigated the causal effect of genetically predicted vitamin D levels on AD risk by deriving a genetic instrument of 59 independent SNPs (r^2^ < 0.001) reported to be most strongly associated with 25-OHD levels in the most recent GWAS (N = 443,734) (Manousaki et al., 2020) (Supplementary Table S2). Summary GWAS data were also available for the most recent AD GWAS meta-analysis (Paternoster et al., 2015). Two-sample MR analysis was performed using the TwoSampleMR R package. A causal estimate was obtained with the inverse-variance weighting method by combining SNP*-*exposure and SNP*-*outcome association in a meta-analysis assuming multiplicative random effects. MR-Egger regression, weighted median analysis, the weighted mode-based estimate, and the MR-Pleiotropy Residual Sum and Outlier method were also performed to investigate potential horizontal pleiotropy, to ensure that the genetic instruments only affect the outcome via the exposure. Horizontal pleiotropy was also investigated by performing Causal Analysis Using Summary Effect Estimates by adopting a whole-genome approach (Morrison et al., 2020). There was little evidence that vitamin D levels causally influence AD risk (OR per SD change in log-transformed 25-OHD levels = 1.233; 95% CI = 0.927–1.639; *P-*value = 0.150), consistent with the causal estimate previously reported (Manousaki et al., 2017).

The causal effect of AD genetic risk on vitamin D levels was also investigated with an AD instrument of 24 SNPs reported in the most current AD GWAS (Paternoster et al., 2015) (Supplementary Table S3). GWAS summary data from the recent vitamin D GWAS were made available by the authors (Manousaki et al., 2020). In the inverse-variance weighting method, there was strong evidence of AD genetic risk causally increasing log-transformed 25-OHD concentrations by 0.043 SD per doubling odds of AD (95% CI = 0.017–0.069; *P-*value = 0.001) (Figure 1 and Supplementary Figure S2). Performing the Steiger directionality test (Hemani et al., 2017) gave evidence that the variance explained by the AD genetic instrument was greater for AD compared with 25-OHD levels (*P*-value < 2.2 × 10^−16^).However, there was evidence of heterogeneity (Cochran Q = 282; *P-*value < 2.2 × 10^−16^) and horizontal pleiotropy (Egger intercept = −0.012; 95% CI = −0.018 to −0.006; *P-*value = 5.4 × 10^−4^) in the causal estimate (Supplementary Figure S1). When correcting for detected pleiotropy with the MR-Pleiotropy Residual Sum and Outlier method, there was still strong evidence of a causal effect on 25-OHD levels (OR = 0.024; 95% CI = 0.012–0.036; *P*-value = 6.0 × 10^−4^). Performing Causal Analysis Using Summary Effect Estimates analysis with AD and 25-OHD GWAS summary data also gave evidence of horizontal pleiotropy (*P-*value_expected log pointwise posterior density_ = 0.13), suggesting that some AD variants influence 25-OHD levels via a shared pathway (Morrison et al., 2020). This appeared to be mostly driven by rs61816761, mapping to the RX501X functional *FLG* mutation strongly associated with AD, which was also found to be strongly associated with 25-OHD levels and other *FLG* mutations including R2447X (rs138726443) and S3247X (rs150597413) (Supplementary Figure S3). Excluding the *FLG* locus from the AD instrument still gave evidence of a causal effect on 25-OHD levels (0.018 SD per doubling odds of AD; 95% CI = 0.004–0.031; *P-*value = 0.008) (Figure 1). There still remained some evidence of heterogeneity (Cochran’s Q = 60, *P-*value = 2.3 × 10^−5^), but there was weak evidence of horizontal pleiotropy (Egger intercept = 0.001; 95% CI = −0.005 to 0.006; *P-*value = 0.782) (Supplementary Figure S4). We explored if the causal relationship detected was limited to AD or was an effect of any inflammatory disease on vitamin D levels. MR analyses were performed to estimate the causal effect of various inflammatory traits on vitamin D levels with genetic instruments (SNPs with *P* < 5 × 10^−8^) derived from individuals of white European ancestry for asthma (Demenais et al., 2018), rheumatoid arthritis (Okada et al., 2014), systemic lupus erythematosus (Bentham et al., 2015), psoriasis (Tsoi et al., 2017), type 1 diabetes mellitus (Onengut-Gumuscu et al., 2015), multiple sclerosis (International Multiple Sclerosis Genetics Consortium et al., 2013), Crohn’s Disease, and ulcerative colitis (de Lange et al., 2017) (Supplementary Tables S1 and S4–S11). The asthma instrument was filtered to exclude SNPs with nominal association with AD (*P* < 0.05). We found some evidence of type 1 diabetes mellitus genetic risk decreasing 25-OHD levels (−0.004 SD per doubling odds of disease; 95% CI = −0.007 to 0.000; *P-*value = 0.044) and evidence of a causal increase with systemic lupus erythematosus genetic risk (0.003 SD per doubling odds of disease; 95% CI = 0.000–0.005; *P-*value = 0.017) (Supplementary Figure S5). Although these estimates do not pass the threshold for multiple testing (0.006), they may warrant further investigation. For the remaining inflammatory traits investigated, there was very little evidence to suggest a causal effect on 25-OHD levels (Supplementary Figure S5).

**Figure 1:**
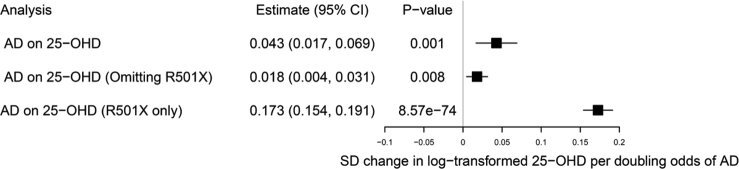
**MR causal estimates of the effect of genetic risk of AD on vitamin D (25-OHD).** An AD instrument comprising all 24 GWAS SNPs, an AD instrument excluding the *FLG* R501X functional mutation, and an AD instrument of the *FLG* R501X functional mutation alone were used. 25-OHD, 25-hydroxycholecalciferol; AD, atopic dermatitis.

A genetic risk score for AD was tested in UK Biobank for association with potential confounders. Regression analyses detected weak associations between the AD genetic risk score and lower body mass index and educational attainment (proxy for socioeconomic status); however, these estimates are of a small magnitude and unlikely to impact the casual effect of AD genetic risk on 25-OHD levels (Supplementary Figure S6).

In this study, we have found evidence that AD is causally associated with an increase in serum vitamin D levels. This is not consistent with observational reports of patients with AD with lower vitamin D levels (Palmer, 2015) and suggests that the true nature of the relationship may have been masked by confounders with opposing effects on vitamin D and AD such as obesity, socioeconomic status, pollution, and latitude (Mesquita et al., 2013). Notably, higher body mass index has been found to be causally associated with increased AD risk but lower 25-OHD levels (Budu-Aggrey et al., 2019; Vimaleswaran et al., 2013). Our findings also provide further evidence that vitamin D supplementation is unlikely to be an effective treatment for AD.

Although the AD-associated *FLG* gene has a particularly strong relationship with vitamin D, other AD SNPs show a consistent direction of effect when omitting this locus, suggesting that AD more generally influences serum vitamin D levels. The substantial heterogeneity and evidence of horizontal pleiotropy observed when including the *FLG* locus in the AD instrument suggests a direct influence of *FLG* on vitamin D that bypasses AD. A link between *FLG* and vitamin D has been previously reported in the same direction that we report (Thyssen et al., 2012). The UVB–vitamin D3 hypothesis provides a proposed mechanism for this, whereby trans-urocanic acid, a breakdown product of FLG, provides epidermal protection against UVB (Mildner et al., 2010). Therefore, inheriting an *FLG*-null mutation (particularly one that reduces histidine in FLG, i.e., the R501X truncation reduces *FLG* from 413 to 35 histidine residues [Fauman, 2020]) would result in increased UV absorption and increased vitamin D3 synthesis. This mechanism has been suggested to be advantageous in northern latitudes, providing an explanation for the latitude-dependent gradients of *FLG* mutation frequency observed (Thyssen and Elias, 2017), and warrants future clinical trials of supplementation or phototherapy to investigate whether different effects are observed in patients with AD with and without *FLG* mutations.

The mechanism explaining the causal influence of AD on vitamin D is unclear. This may be explained by biological effects in the skin similar to those seen for *FLG* or behavioral mechanisms that may alter UV and/or vitamin exposure in AD sufferers. The vitamin D GWAS adjusted for vitamin D supplementation (Manousaki et al., 2020) but may have included individuals with AD whose vitamin D status was raised because of vitamin D–raising therapy. However, as the UK Biobank showcase indicates that only 69 of 410,256 participants were recorded to have received phototherapy, this is unlikely to explain our findings.

In this study, we confirm that there is weak evidence that vitamin D causally influences AD. Our findings suggest that vitamin D supplementation is not supported as an AD therapy. Further investigation is required to determine the mechanisms of the causal effect of AD genetic risk on serum vitamin D levels. Stratifying future vitamin D and phototherapy trials and epidemiological analyses by *FLG* status may assist in evaluating the contribution of different mechanisms.

### Data availability statement

Datasets related to this article can be found at https://github.com/abudu-aggrey/Atopic_Dermatitis_25OHD_MR. Summary GWAS data for vitamin D (25-OHD) was made available by the authors on request (Manousaki et al., 2020).

## ORCIDs

Daniel R. Drodge: http://orcid.org/0000-0002-2733-8509

Ashley Budu-Aggrey: http://orcid.org/0000-0002-8911-2492

Lavinia Paternoster: http://orcid.org/0000-0003-2514-0889

## Conflict of Interest

LP has received personal fees from Merck for Scientific Input Engagement related to Mendelian randomization methodology. The remaining authors state no conflict of interest.
