# Supplementary material for: Causal Analysis Shows Evidence of Atopic Dermatitis Leading to an Increase in Vitamin D Levels
Source: J Invest Dermatol. 2021 May;141(5):1339–41. doi: 10.1016/j.jid.2020.09.013 (PMC8050017; doi:10.1016/j.jid.2020.09.013)
Supplement: Supplementary Information [file mmc1.pdf]

Supplementary Material

Causal analysis shows evidence of atopic dermatitis leading to an increase in vitamin D levels, Drodge DR et al

Supplementary Tables

Tables S1 to S11
Available online at https://github.com/abudu-aggrey/Atopic_Dermatitis_25OHD_MR

Supplementary Figures

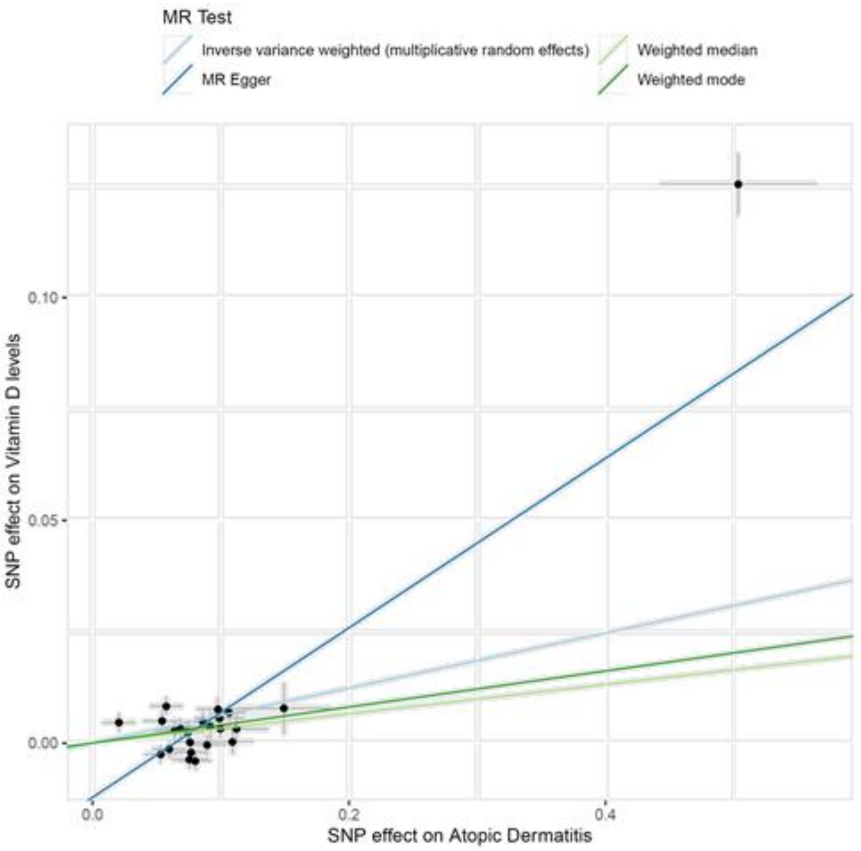

Figure S1 – MR Egger Plot for causal effect of liability for Atopic Dermatitis (AD) upon serum vitamin D levels. Association of AD SNPs with AD (ln(OR)) and serum vitamin D levels (beta) are displayed. MR Egger Estimate: 0.190 (95% CI = 0.122 to 0.259); weighted median estimate: 0.033 (95% CI = 0.001 to 0.013); weighted mode estimate: 0.040 (95% CI = 0.044 to 0.003); Egger intercept: -0.012 (95% CI = -0.018 to -0.006); MR-PRESSO Estimate: 0.035 (95% CI = 0.018 to 0.052); Q statistic: 282 (*P*-value< 2.2×10^{-16}).

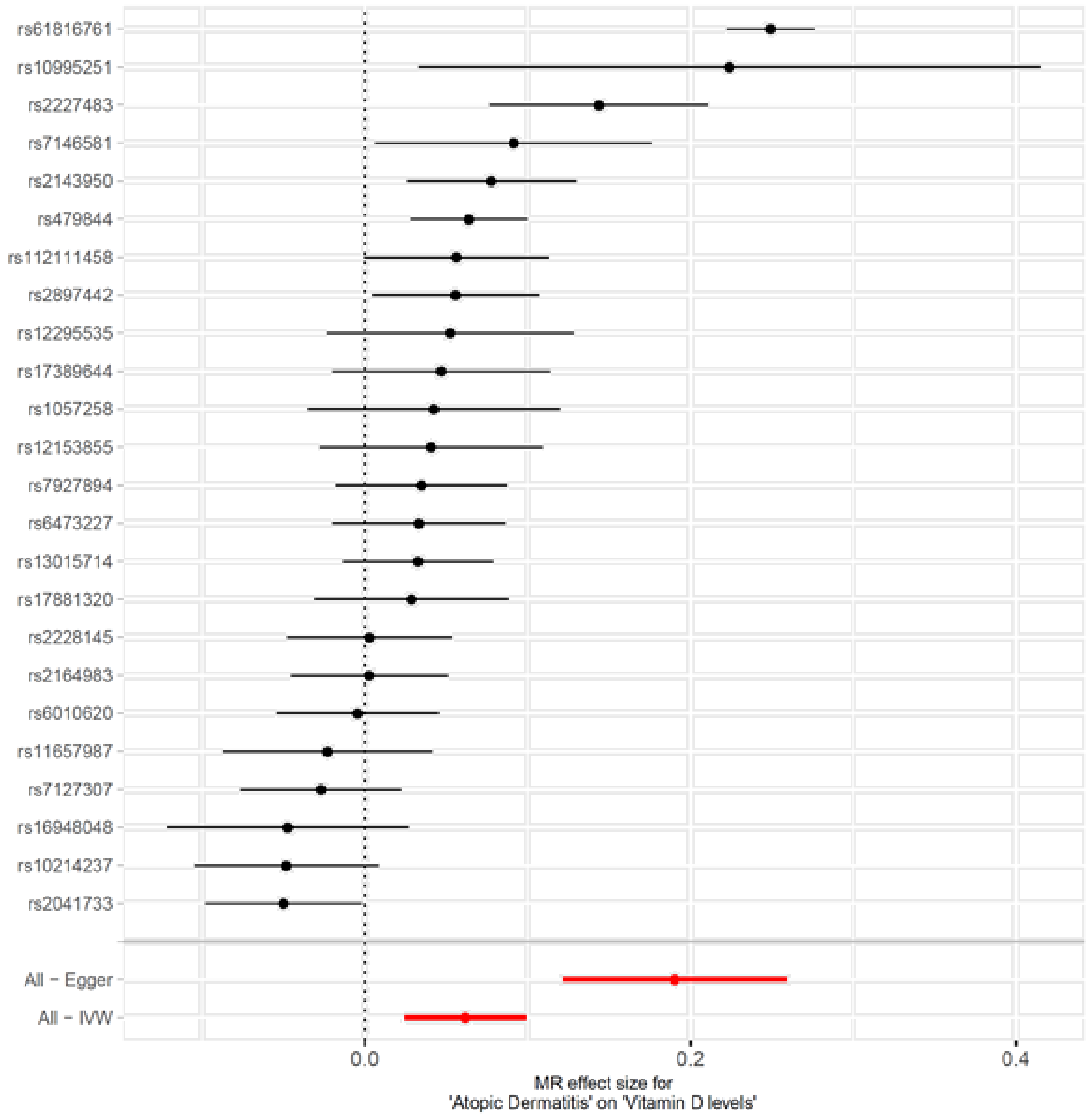

Figure S2: **Causal effect of liability for Atopic Dermatitis (AD) upon serum vitamin D levels.** Effect estimates are displayed for each SNP within the AD instrument. Estimates given for SD change log-transformed 25-OHD per logs odds of AD risk.

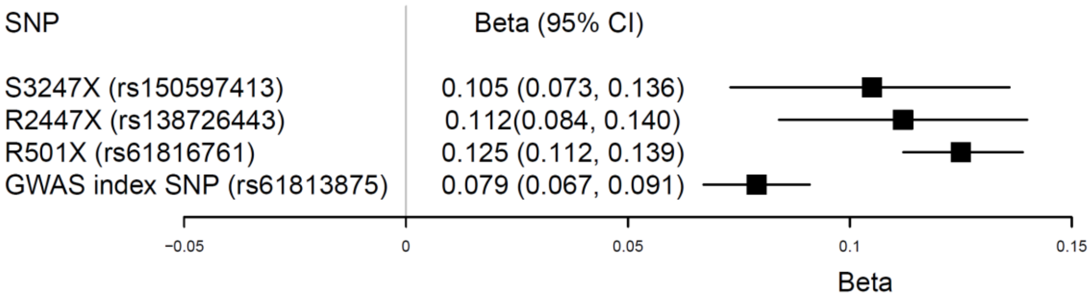

Figure S3: Association between FLG variants and serum vitamin D levels. First three variants are established functional FLG mutations, rs61813875 is the index GWAS SNP from this region, identified to tag R501X (Paternoster et al, 2015). Beta represents standard deviation increase of standardised log-transformed 25-OHD levels per allele. CI, confidence interval

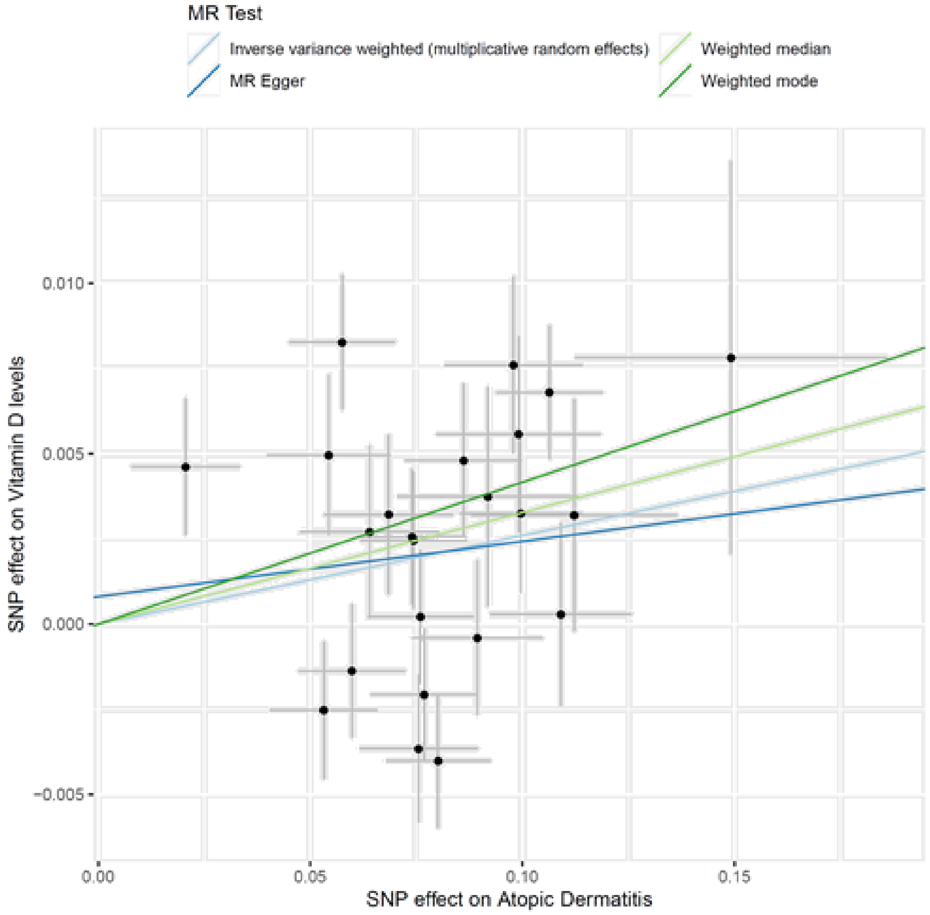

Figure S4 – MR Egger Plot for causal effect of liability for Atopic Dermatitis (AD) upon serum vitamin D levels, excluding R501X (rs61816761) from the AD instrument. Association of AD SNPs with AD (ln(OR)) and serum vitamin D levels (beta) are displayed. MR Egger Estimate: 0.016 (95% CI = -0.055 to 0.087); weighted median estimate: 0.033 (95% CI = 0.013 to 0.052); weighted mode estimate: 0.042 (95% CI = 0.003 to 0.080); Egger intercept: 0.001 (95% CI = -0.005 to 0.006); MR-PRESSO: 0.184 (95% CI = -0.129 to 0.496); Q statistic: 60 (*P*-value = $2.33x10^{-5}$).

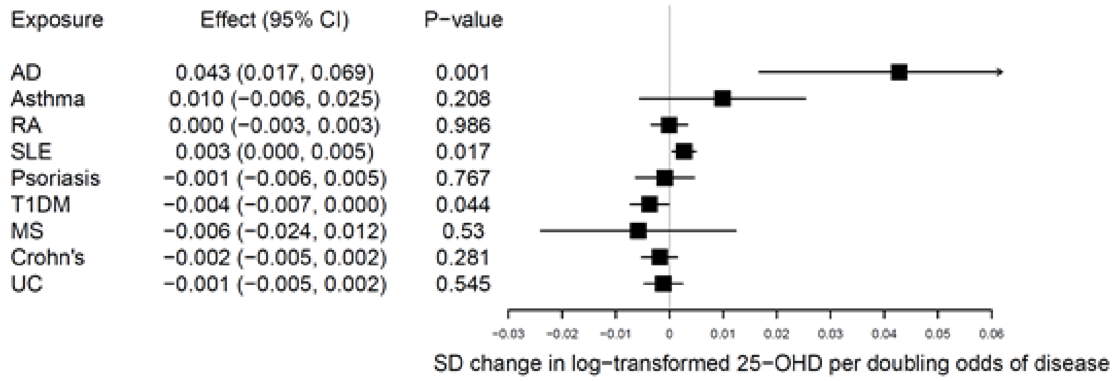

Figure S5 - Causal effect estimates of various exposure disease states on serum 25-OHD expressed as standard deviation (SD) change in log 25-OHD per doubling odds of disease, as estimated using Mendelian Randomization. AD: atopic dermatitis; RA: rheumatoid arthritis; SLE: systemic lupus erythematosus; T1DM: type 1 diabetes mellitus; MS: multiple sclerosis; UC: ulcerative colitis.

Association analyses with genetic risk scores

A genetic risk score for atopic dermatitis (AD) was created in UK Biobank with SNPs reported to be most strongly associated (Paternoster et al, 2015). The risk score was standardised to have a mean of 0 and standard deviation of 1. Regression analyses were performed with BMI and educational attainment (proxy for socioeconomic status) to investigate associations with potential confounders of the AD-25-OHD relationship within UK Biobank. The BMI (kg/m^2) of UK Biobank participants was calculated from height and weight measurements. Educational attainment was defined by responses to a touchscreen questionnaire where participants were asked “which of the following qualifications do you have?”, where participants could select more than one option including “College or University degree”, “A levels/AS levels or equivalent”, “O levels/GCSEs or equivalent”, “CESs or equivalent”, “NVQ or HND or HNC or equivalent”, “Other professional qualifications eg: nursing, teaching”, or “None of the above”. Participant responses were coded into categorical variables for degree holders, those who had completed advanced level studies (A-level) or had obtained their general certificate of secondary education (GCSE). Analyses was performed while controlling for sex and genotyping chip.

a)

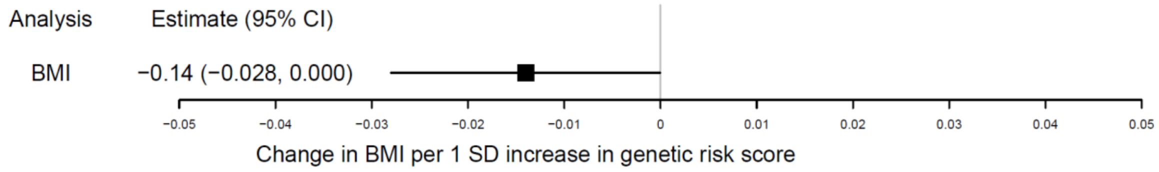

b)

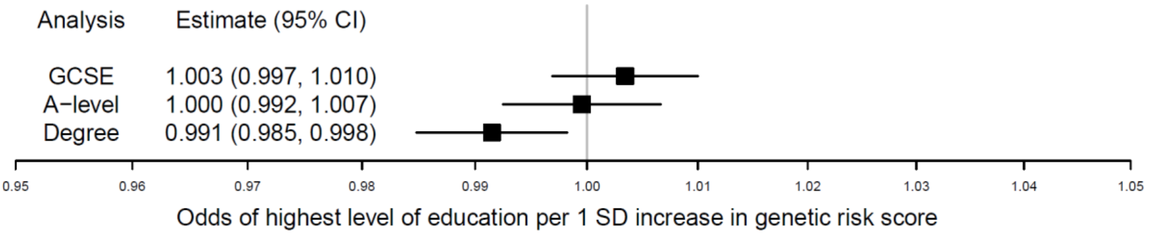

Figure S6 – Association of genetic risk score (GRS) for AD with (a) BMI and (b) highest level of education in UK Biobank (n= 463,005). A-level, Advanced level studies; CI, confidence interval; GCSE, General Certificate of Secondary Education
